# Healthcare Utilization and Expenditures among Iranian Chemical Warfare Survivors Exposed to Sulfur Mustard

**DOI:** 10.34172/aim.2022.40

**Published:** 2022-04-01

**Authors:** Parisa Mehdizadeh, Mostafa Ghanei, Abolghasem Pourreza, Ali Akbari-sari, Batool Mousavi, Rajabali Darroudi

**Affiliations:** ^1^Department of Health Management and Economics, School of Public Health, Tehran University of Medical Sciences, Tehran, Iran; ^2^Health Management Research Center, Baqiyatallah University of Medical Sciences, Tehran, Iran; ^3^Chemical Injuries Research Center, Baqiyatallah University of Medical Sciences, Tehran, Iran; ^4^Prevention Department, Janbazan Medical and Engineering Research Center (JMERC), Tehran, Iran

**Keywords:** Chemical warfare, Expenditure, Health care, Utilization

## Abstract

**Background:**

This study aims to explore the healthcare utilization (HCU) and expenditures from complementary health insurance among Iranian people who survived sulfur mustard (SM) exposure during the Iran-Iraq war.

**Methods:**

This study was conducted between March 21, 2018 and March 21, 2019 using secondary data. Data on the HCU and expenditure of war survivors after their exposure to SM were obtained from the Iran Veterans and Martyr Affair Foundation (VMAF) database and the national complementary insurance organization that covers their medical expenses. Multiple linear and zero-inflated poison regression (ZIP) models were used to estimate the costs and HCU. Analyses were performed in R software version 3.6.3.

**Results:**

Among 58880 survivors who were included in study, 36383 (61.7%) used at least one service during a year. The total frequency of HCU was 15.6 services per person per year. The annual mean medical cost of each survivor was US$807.6 (±2901.2). The highest number of utilizations was related to medicine and physician visits. The highest median cost was related to rehabilitation (US$151.7), medical equipment (US$84.5), medicine (US$83.3) and inpatient services (US$ 48.8). With increasing age, disability, weight, severity of injury in lung or eye injuries, the rate of health service utilization rose significantly.

**Conclusion:**

Over 30 years after the Iran-Iraq war, Iranian people who were exposed to SM and survived still suffer from injuries and pose a significant drain on healthcare resources.

## Introduction

 War and its consequences threaten human health even years after the end and may have many direct and indirect effects on the rate of human mortality.^1,2^ Chemical weapons are one of the most inhumane man-made tools of warfare that cause disorders in the human body due to their severe and chronic effects.^3^ Chemical weapons include various types, and nerve agents and sulfur mustard (SM) could be considered as the most well-known among them.^4^ The first use of this kind of weapons after World War II dates back to the Iran-Iraq war in the 1980s.^5^ In the Iraq-Iran war (1980–1988) chemical weapons were used several times against Iranian civilians and soldiers, resulting in deaths and > 45 000 victims of delayed complications.^5,6^ Amini et al reported that 64 190 chemical survivors were registered in the Iran Veterans and Martyr Affair Foundation (VMAF) database.^7^

 The studies conducted to date show that SM has different effects with varying severity on three vital organs; the lung, skin and eyes.^6,8^ In the acute phase of exposure, it could cause mild conjunctivitis in the eye, skin and pulmonary system.^8^ In the delayed and chronic phase of SM exposure, the victims may suffer from blindness, obstruction of upper airways, chronic bronchitis, and bronchiolitis in the lung and many known or unknown disorders.^8,9^ If these lesions are severe enough, they will increase the chance of mortality. The late consequences of SM may occur even up to 50 years after exposure.^3^

 Now, 30 years after the end of the war, chemical warfare survivors are suffering from many physical and mental injuries. There are few studies about the utilization of medical/health services among chemical warfare survivors.^10,11^ There has been no inclusive study on the healthcare utilization (HCU) and costs of HC in chemical warfare injuries. On the other hand, the medical costs imposed due to chemical warfare exposure are expected to increase over time. The medical expenses of these patients may increase due to frequent need for hospitalizations, diagnostic tests (clinical, laboratory, imaging), continuous medical treatment, multiple surgical procedures, psychotherapy, rehabilitation, alternative therapies, specialized counseling and other social care, education, research, etc.^12^ But there is not enough evidence about the amount of costs and utilization of health/medical care among this population who were exposed to chemical warfare, especially SM, over time.

 Bischoff et al, who aimed to determine the cost of HC for war refugees, found that HC costs in war-torn countries were twice as high as other countries.^13^ More than a billion people, mostly in low- and middle-income countries, do not have access to the HC they need for a variety of reasons.^14^ Governments should play a major role in providing HC to the population, but only a small portion of government’s revenue is allocated to this purpose. In Iran, the government allocates an adequate part of its budget to HC expenses for war survivors and their families. Providing insurance coverage to the war survivors’ community and covering most HC expenses is the policy of VMAF. Insurance coverage includes health services, physician visits, outpatient/inpatient surgeries, medications, paraclinical services, nursing, refractive errors treatment, ambulance transport (in or between the cities) and home care services.^15^

 In Iran, since 2000, all war survivors and their families and also martyrs’ families are covered by complementary health insurance. Most of their medical and pharmaceutical expenses are covered by basic and complementary health insurance. VMAF has a contract with one of the private insurance companies, based on a per capita monthly payment system in which the insurance companies are committed to covering medical expenses and surgeries subject to insurance in excess of the basic insurer.^16^

 Due to the limited existing studies conducted on HCU of chemical warfare survivors, this research covers a very large and sufficient sample size and a set of important variables that will help in explaining the HCU and its costs in chemical warfare survivors who use complementary health insurance.

## Materials and Methods

###  Study Design 

 A nationwide, registry-based and cross-sectional study was conducted using secondary data sources to measure HCU and HC expenditure over a period of one year (from March 21, 2018 until March 21, 2019) among chemical warfare survivors who were exposed to SM.

 The data of all war survivors registered in the VMAF and their HC services are mostly free of charge. All injured survivors have to register in VMAF in order to receive free HC, and their insurance continues to provide the highest quality of long-term healthcare to war survivors and their families.^17^ The institute’s headline duties include securing pensions, health insurance coverage, providing rehabilitation services, a medical commission consisting of physicians and specialists to periodically examine and record related complications, providing habitation, employment support, social-cultural and sports services, establishment of a research center, and providing nursing home services.

 In addition to the VMAF database, a complementary health insurance database was used for medical and HCU. VMAF has contracts with two health insurance companies, one for basic health insurance and another for complementary insurance. Complementary insurance covers surplus medical services which are not covered by basic health insurance (social health insurance). There is a contract between VMAF and the insurance company to cover health/medical expenditures in a per capita payment system.^18^ These data sources include all HC and their expenditure for war survivors and their families. In this study, only the data of survivors (as head of households) were extracted during one year from March 2018 through March 2019. These services include visits, medical, paraclinical, diagnostic, pharmaceutical, inpatient services, nursing, screening, emergency care, rehabilitation, etc. In this research, each dollar was considered equal to currency exchange rate at the time of payments as 42 000 Rials.^19^

###  Inclusion/Exclusion Criteria

 All data of the survivors exposed to SM were included. Only the HCU of survivors was examined and the data of their families were excluded from the study.

###  Study Sample and Data Collection

 Among all SM exposed survivors (n = 64 190 based on Amini et al),^7^ who were available and registered in the VMAF database, in the present study, the research population was 58,880 who were alive during the whole or part of study period.

 In the VMAF database, all war-related types of injuries are recorded based on injuries and severity of lesions in lung, eyes and skin.^7^ Data related to the injuries caused by chemical warfare were classified in the VMAF database by the involved organs, disability weights and the severity of late complications including mild, moderate and severe. The diagnosis of the injuries and severity of the lesions in lung, eyes and skin, were determined by a medical experts panel.^3^ The members of this panel included pulmonologists, psychiatrists, neurologists, ophthalmologists, dermatologists, cardiologists, forensic medicine experts and nephrologists. Expert panel members investigate the victim’s documents case by case and record the associated complications of chemical warfare injuries; the assessment process is done constantly.

 Excel sheets was prepared for the variables of the study which include sex, age, residence area, type of injury (lung, eye, skin), severity of the injuries (mild, moderate and severe), disability weight (based on the severity of the injury and symptomatic nature of the acute and long-term complications, each survivor will receive a weighted disability score between 5 and 70 (the more severe the injury, the higher the disability weight), health and medical utilization and its costs. The source of this file was two databanks from VMAF and complementary insurance system that register all HCU and costs for all survivors and their families. After extracting and merging the variables from these data sources, duplicate data were removed and finally the cleaned data were entered into a single data bank.

###  Data Analysis

 In addition to descriptive information including frequency, percentage and median, first and third quartile, interquartile range, mean and standard deviation, the purpose of the study was to evaluate the effect of age categories, disability weight, and severity (lungs, eyes, and skin) on the average number of services received per year per person and the total annual cost of services per person.

 For the number of services received, because there was a significant percentage of zero in the number of services, the Zero-inflated poison regression (ZIP) model was used. This model contains two parts, extra zero and count. To investigate the covariates’ effect on receiving or not receiving services (zero part) and the count of service (count part), covariates (age categories, disability weight, lung severity, eye severity, skin severity) to parts separately. The details of the model are described below.


$$P\left(y_{i}=j\right)=\left\{\begin{array}{l} \pi_{i}+\left(1-\pi_{i}\right)e^{-\mu_{i}}\;if\;j=0\\ \left(1-\pi_{i}\right)\frac{\mu_{i}^{y_{i}}e^{-\mu_{i}}}{y_{i}!}\;if\;j>0 \end{array}\right.$$

$$\mu_{i}=e^{{\mathrm{cov}}_{i}}$$

$$\pi_{i}=\frac{\lambda_{i}}{1+\lambda_{i}}$$

$$\lambda_{i}=e^{zero_{i}}$$

$${\mathrm{cov}}_{i}=\beta_{o}+\beta_{1}A2_{i}+\beta_{2}A3_{i}+\beta_{3}A4_{i}+\beta_{4}L2_{i}+\beta_{5}L3_{i}+\beta_{6}L4_{i}+\beta_{7}S2_{i}+\beta_{8}S3_{i}+\beta_{9}S4_{i}+\beta_{10}E2_{i}+\beta_{11}E3_{i}+\beta_{12}E4_{i}+\beta_{13}D2_{i}+\beta_{14}D3_{i}$$

where:

- *y*_i_ is the number of services received for the i^th^ person
- *π*_i_ is the probability of extra zeros for the i^th^ person
- *cov*_i_ is the independent variables term for the i^th^ person
- *β*_0_ is the intercept
- If the age category of the i^th^ veteran is 50-59, the *A2*_i_ variable is equal to one; otherwise, it is equal to zero.
- If the age category of the i^th^ veteran is 60-69, the *A3*_i_ variable is equal to one; otherwise, it is equal to zero.
- If the age category of the i^th^ veteran is 70 + , the *A4*_i_ variable is equal to one; otherwise, it is equal to zero.
- If the lung severity of the i^th^ veteran is mild, the *L2*_i_ variable is equal to one; otherwise, it is equal to zero.
- If the lung severity of the i^th^ veteran is moderate, the *L3*_i_ variable is equal to one; otherwise, it is equal to zero.
- If the lung severity of the i^th^ veteran is severe, the *L4*_i_ variable is equal to one; otherwise, it is equal to zero.
- If the eye severity of the i^th^ veteran is mild, the *E2*_i_ variable is equal to one; otherwise, it is equal to zero.
- If the eye severity of the i^th^ veteran is moderate, the *E3*_i_ variable is equal to one; otherwise, it is equal to zero.
- If the eye severity of the i^th^ veteran is severe, the *E4*_i_ variable is equal to one; otherwise, it is equal to zero.
- If the skin severity of the i^th^ veteran is mild, the *S2*_i_ variable is equal to one; otherwise, it is equal to zero.
- If the skin severity of the i^th^ veteran is moderate, the *S3*_i_ variable is equal to one; otherwise, it is equal to zero.
- If the skin severity of the i^th^ veteran is severe, the *S4*_i_ variable is equal to one; otherwise, it is equal to zero.
- If the disability weight of the i^th^ veteran is 25-49, the *D2*_i_ variable is equal to one; otherwise, it is equal to zero.
- If the disability weight of the i^th^ veteran is 50-70, the *D3*_i_ variable is equal to one; otherwise, it is equal to zero.
- *zero*_i_ is the independent variables term for the extra zero part of the model, for the i^th^ person. The formula is the same as the *cov*_i_, with the difference that all the coefficients in the*zero*_i_ are estimated independently and separately.

After logarithmic transformation on cost value, multiple linear regression models were used for the cost of services. The covariates used are the same as the ZIP model. The details of the model are described below.


$$\begin{array}{l} \log_{e}\left(y_{i}\right)={\mathrm{cov}}_{i}+\varepsilon \\ \varepsilon ∼Normal\left(0,\delta \right) \end{array}$$

where:

- *y*_i_ is the total annual cost of services for the i^th^ person
- *cov*_i_ is the independent variables term for the i^th^ person. The formula is the same as *cov*_i_ described in the ZIP model.
- *ε* is the error term.

All analyses were performed in R software version 3.6.3.

## Results

 Most of the study’s population were in the 50–59 years age group (72.49%) and male (97.98%). Table 1 shows the demographic and clinical characteristics of the study population. About two-thirds (36 383; 61.7%) of them used at least one service during a year. Chi-square test was performed between age categories and study population, utilized HC, categories of severity lung, categories of severity eye, categories of severity skin, joint complication and in all cases *P* value was less than 0.001. Similarly, instead of age groups, the test was performed for gender, and the *P* value was less than 0.001.

**Table 1:** Demographic and Clinical Characteristics of Studied Population

**Category**	**Group**	**Age**				**Gender**	
		**<50**	**50 – 59**	**60 – 69**	**70+**	**Male**	**Female**
Study population (*n* = 58880)		3457	42683	9838	2902	57693	1187
Utilized HC (*n* = 36383)		2509	26253	5701	1920	35302	1081
Lung	No lesions (*n* = 28849)	1650 (47.8%)	21314 (49.9%)	4567 (46.4%)	1318 (45.5%)	28377 (49.2%)	472 (39.8%)
	Mild (*n* = 24086)	1500 (43.4%)	17354 (40.7%)	4093 (41.6%)	1139 (39.2%)	23490 (40.7%)	596 (50.2%)
	Moderate (*n* = 5563)	292 (8.4%)	3773 (8.8%)	1089 (11.1%)	409 (14.1%)	5450 (9.4%)	113 (9.5%)
	Severe (*n* = 382)	15 (0.4%)	242 (0.6%)	89 (0.9%)	36 (1.2%)	376 (0.7%)	6 (0.5%)
Eye	No lesions (*n* = 50911)	3076 (89%)	36808 (86.2%)	8552 (86.9%)	2475 (85.3%)	49835 (86.3%)	1076 (90.6%)
	Mild (*n* = 7641)	367 (10.6%)	5636 (13.2%)	1229 (12.5%)	409 (14.1%)	7533 (13.1%)	108 (9.1%)
	Moderate (*n* = 159)	6 (0.2%)	108 (0.3%)	35 (0.4%)	10 (0.3%)	156 (0.3%)	3 (0.3%)
	Severe (*n* = 169)	8 (0.2%)	131 (0.3%)	22 (0.2%)	8 (0.3%)	169 (0.3%)	0 (0%)
Skin	No lesions (*n* = 52836)	3071 (88.9%)	38476 (90.1%)	8742 (88.9%)	2547 (87.7%)	51868 (89.9%)	968 (81.6%)
	Mild (*n* = 5389)	322 (9.3%)	3779 (8.9%)	973 (9.9%)	315 (10.9%)	5212 (9%)	177 (14.9%)
	Moderate (*n* = 561)	52 (1.5%)	369 (0.9%)	109 (1.1%)	31 (1.1%)	526 (0.9%)	35 (2.9%)
	Severe (*n* = 94)	12 (0.3%)	59 (0.1%)	14 (0.1%)	9 (0.3%)	87 (0.2%)	7 (0.6%)
Joint complication*	Lung & Eye & Skin (*n* = 1236)	90 (19.7%)	883 (15.7%)	188 (13.2%)	75 (15.2%)	1187 (15.3%)	49 (23%)
	Skin & Eye (*n* = 467)	24 (5.3%)	336 (6%)	80 (5.6%)	27 (5.5%)	457 (5.9%)	10 (4.7%)
	Skin & Lung (*n* = 2953)	193 (42.2%)	1999 (35.5%)	575 (40.6%)	186 (37.8%)	2835 (36.4%)	118 (55.4%)
	Eye & Lung (*n* = 3338)	150 (32.8%)	2409 (42.8%)	575 (40.6%)	204 (41.5%)	3302 (42.4%)	36 (16.9%)

*The lines show descriptions of survivors who have all three complications, who have eye and skin complications but no lung complications, have both skin and lung complications but no eye complications, and have both eye and lung complications but no skin complications. A complication here means that one of the three levels of injury severity has occurred (mild, moderate, or severe).

Table 2 shows the total HCU, total annualized number of HCU and HC costs of the studied population. HCU and indicators (outpatient and inpatient) in the study population who were under the coverage of complementary insurance are shown in Table 2 (N = 58 880).

**Table 2:** Distribution of Health Care Utilization and Costs (US$) in SM Exposed Survivors (N = 58880)

**Type of Cost**	**Annualized HC Utilization**	**Cost US$ (IRR)**						
		**Median**	**Q1**	**Q3**	**Interquartile Range**	**Mean**	**Standard Deviation**	**Total Cost (%)**
Paraclinic	1.06	53.9 (2263800)	22.6 (949200)	136.6 (5737200)	113.9 (4783800)	128.3 (5388600)	449.8 (18891600)	2667376 (112029792000) (9.1%)
Outpatient	14.6	180.5 (7581000)	62.2 (2612400)	460.5 (19341000)	398.4 (16732800)	607.6 (25519200)	2471 (103782000)	21841728 (917352576000) (74.3%)
- Medicine	6.52	83.3 (3497821)	28.2 (1182512)	232.8 (9778140)	204.7 (8595627)	412.3 (17315279)	2075.5 (87170320)	334694 (14057148000) (1.1%)
- Physician visit	6.14	54.1 (2274300)	21.8 (916975)	115.8 (4862005)	93.9 (3945030)	130.7 (5489397)	916.6 (38495353)	2191728 (92052576000) (7.5%)
- Emergency	0.76	7.5 (7581000)	3.1 (130200)	20.3 (852600)	17.2 (722400)	28.2 (1184400)	157.4 (6610800)	53432 (2244144000) (0.2%)
- Imaging	0.67	38.1 (1600000)	17.3 (727050)	84.3 (3539702)	67(2812652)	126.3 (5302569)	572 (24024222)	171873 (7218666000) (0.6%)
- Laboratory	0.025	38.9 (1635800)	24.1 (1014000)	54.3 (2280616)	30.2 (1266616)	48.7 (2045717)	46.1 (1936165)	13966009 (586572378000) (47.5%)
- Nursing	0.36	5.2 (216640)	3.6 (150000)	10.9 (456280)	7.3 (306280)	17.7 (741976)	98.9 (4155459)	810765 (34052130000) (2.8%)
- Rehabilitation	0.11	151.7 (6370920)	83.3 (3500000)	265 (11129510)	181.7 (7629510)	231 (9701458)	318.7 (13385004)	27838 (1169196000) (0.1%)
- Screening	0.025	23.8 (1000000)	19 (800000)	47.6 (2000000)	28.6 (1200000)	35.8 (1504747)	28.8 (1207821)	4285389 (179986338000) (14.6%)
Hospitalization/inpatient	0.93	48.8 (2050463)	25.7 (1080430)	103.6 (4351601)	77.9 (3271171)	318.4 (13372901)	1500.2 (63006923)	7077449 (297252858000) (24.1%)
Medical equipment	0.015	84.5 (3550000)	25.1 (1053000)	235.1 (9875000)	210 (8822000)	501.6 (21068689)	1622.4 (68142805)	329574 (13842108000) (1.1%)
Other	0.044	19 (798000)	7.1 (298200)	54 (2268000)	46.8 (1965600)	85.8 (3603600)	414.1 (17392200)	132935 (5583270000) (0.5%)
**Total cost**	15.6	224.7	80	597.9	517.9	807.6	2901.2	29381687 (100)

The number of services used by SM exposed survivors was 915,858 (15.6 annual HCU). The median total cost was US$224.7 and the mean HC cost per person per year was US$807.6 (SD = 2901.2). The highest number of HCU was related to medicine and physician visits. Among all, the highest cost pertained to rehabilitation (US$151.7), medical equipment (US$84.5), medicine (US$83.3) and inpatient services (US$48.8). Furthermore, 74.3% of total costs were related to outpatient and 24.1% were related to inpatient services.

Table 3 shows the determinants of HCU in SM exposed survivors. In the count column in Table 3, the results related to the count of HCU in SM exposed survivors are presented. With increasing age, the severity of injury in survivors with lung and eye injuries, and disability weight (with the stability of other variables), the expected number of HCU per person increased, but it decreased in the case of skin. For example, the expected number of HCU per person for severe lung injury is 1.134 times greater than for a “No lesions” level of severity when all other variables in the model are constant. In other words, under the same conditions, they receive 13.4% more HC per year than the base group (No lesions).

**Table 3:** Determinants of Health Care Utilization in SM Exposed Survivors

**Category**	**Group**	**Number**	**No. of HCU**	**% of HCU**	**Total Number of HCU in Year**	**Annualized HCU Rate**	**Count** **RR (** ***P*** ** Value)**	**Zero** **OR(** ***P*** ** Value)**
Age	< 50	3457	2509	72.6	54477	15.8	Ref	Ref
	50 – 59	42683	26253	61.5	629965	14.8	1.067 (< 0.001)	1.294 (< 0.001)
	60 – 69	9838	5701	57.9	170814	17.4	1.329 (< 0.001)	1.589 (< 0.001)
	70 +	2902	1920	66.2	60602	21.2	1.377 (< 0.001)	1.208 (0.001)
Lung	No lesions	28849	17182	59.6	403813	14	Ref	Ref
	Mild	24086	15183	63	394379	16.4	1.056 (< 0.001)	0.874 (< 0.001)
	moderate	5563	3747	67.4	108204	19.5	1.053 (< 0.001)	0.708 (< 0.001)
	Severe	382	271	70.9	9462	25	1.134 (< 0.001)	0.522 (< 0.001)
Eye	No lesions	50911	31211	61.3	776752	15.3	Ref	Ref
	Mild	7641	4934	64.6	131485	17.3	1.017 (< 0.001)	0.787 (< 0.001)
	moderate	159	114	71.7	3015	19	0.883 (< 0.001)	0.652 (0.02)
	Severe	169	124	73.4	4606	27.4	1.175 (< 0.001)	0.485 (< 0.001)
Skin	No lesions	52836	32366	61.3	811499	15.4	Ref	Ref
	Mild	5389	3574	66.3	92583	17.2	0.933 (< 0.001)	0.858 (< 0.001)
	moderate	561	371	66.1	10129	18.1	0.926 (< 0.001)	0.929 (0.436)
	Severe	94	72	76.6	1647	17.6	0.75 (< 0.001)	0.608 (0.049)
Disability weight	0 – 24	34225	21315	62.3	486539	14.3	Ref	Ref
	25 – 49	21970	13489	61.4	377244	17.2	1.202 (< 0.001)	1.079 (< 0.001)
	50 – 70	2685	1579	58.8	52075	19.5	1.375 (< 0.001)	1.196 (< 0.001)
Overall		58880	36383	61.8	915858	15.6		

HCU, health care utilization; RR, risk ratio; OR, odds ratio

 In the Zero column in Table 3, the results related to the odds of not using HC in SM exposed survivors are presented. With increasing disability weight (with the stability of other variables), the expected odds of not using HC per person increased, but it decreased in survivors with lung and eye. For example, in age groups, the odds of not using HC services for the age group of 70 + , was 1.208 times than the base age category (< 50 years) when all other variables in the model were constant. For lung injuries, for those in the high level of severity, the odds of not using HC was 0.522 compared to base severity (No lesions). In other words, with increasing severity of lung, there was an increase in the utilization of HC.

Table 4 shows the annualized HC costs of SM exposed survivors and their determinants. According to the results, with increasing age, the severity of injury in lung and disability weight, the average annual cost of HCU increased. For people in the age group of 70 + , the average annual cost of HCU per person was 2.0 times greater than the base age group (< 50 years). In other words, the average annual cost of HCU in the age group of 70 + was 104.7% higher than the base group. In survivors, increasing severity of injury was equal to a considerable rising in the average annual cost of HCU and severe lung injuries were significantly associated with higher costs compared to other injuries.

**Table 4:** Annualized Health Care Cost and its Determinants in SM Exposed Survivors

**Category**	**Group**	**Total Payment US$ (IRR)**						
		**Median**	**Q1**	**Q3**	**Interquartile Range**	**Mean**	**Standard** **Deviation**	**Coefficient** **(** ***P*** **-Value)**
Age	< 50	188.6 (7922672)	67.9 (2851071)	450.1 (18904794)	382.2 (16053723)	616.8 (25904458)	2417.2 (101524177)	ref
	50-59	204.5 (8589000)	73.7 (3096588)	543.6 (22832966)	469.9 (19736378)	744 (31247140)	2697.1 (113279283)	1.106 (0.003)
	60-69	308.1 (12941608)	106.6 (4478790)	827.7 (34762964)	721.1 (30284174)	1041.6 (43745454)	3728.7 (156607240)	1.588 (< 0.001)
	70 +	416.8 (17504522)	141.7 (5951018)	1030.6 (43284029)	888.9 (37333011)	1231.6 (51725297)	3268.8 (137291032)	2.047 (< 0.001)
Lung	No lesions	188.1 (7898578)	66.8 (2807200)	501.2 (21048940)	434.3 (18241740)	738.5 (31016902)	3263.7 (137074756)	ref
	Mild	251.3 (10553090)	88.9 (3735494)	649.1 (27260961)	560.1 (23525466)	819.8 (34431604)	2485.7 (104399359)	1.2 (< 0.001)
	Moderate	306.6 (12877560)	110.8 (4652424)	841.5 (35344877)	730.8 (30692454)	1018.3 (42768265)	2684.8 (112761307)	1.225 (< 0.001)
	Severe	621.9 (26120823)	178.9 (7515782)	1919.5 (80617302)	1740.5 (73101520)	1587.7 (66681582)	2725.9 (114487916)	1.508 (< 0.001)
Eye	No lesions	217.8 (9149130)	77.7 (3264895)	576.8 (24225949)	499.1 (20961054)	787.1 (33056862)	2965.2 (124538225)	ref
	Mild	264.6 (11114547)	92 (3863208)	725.2 (30457638)	633.2 (26594429)	929.2 (39027870)	2513.5 (105566462)	1.15 (< 0.001)
	Moderate	243.5 (10227997)	78.3 (3288004)	651.2 (27351640)	572.9 (24063636)	790.4 (33198484)	1645.8 (69121893)	0.783 (0.103)
	Severe	636.7 (26741123)	226.5 (9511590)	1385.3 (58180727)	1158.8 (48669137)	1141.5 (47943572)	1434 (60226509)	1.399 (0.024)
Skin	No lesions	222.8 (9358224)	79.6 (3341158)	589 (24736206)	509.4 (21395048)	802.1 (33688922)	2946.2 (123740548)	ref
	Mild	240.6 (10106276)	82.6 (3469739)	648.3 (27226827)	565.6 (23757088)	835.9 (35107809)	2491.7 (104652749)	0.855 (< 0.001)
	Moderate	278.2 (11686250)	114.3 (4802494)	814.5 (34208106)	700.1 (29405613)	998.5 (41936138)	2780.9 (116797922)	0.915 (0.29)
	Severe	297.1 (12478934)	84.5 (3549497)	604.7 (25396268)	520.2 (21846772)	866.9 (36410762)	1836.6 (77138450)	0.735 (0.103)
Disability weight	0-24	191.8 (8057162)	68.3 (2866875)	498.6 (20941761)	430.4 (18074886)	666.4 (27986794)	2460.2 (103326323)	ref
	25-49	269.2 (11308116)	97.1 (4076112)	716.1 (30075277)	619 (25999165)	936.6 (39338977)	3385.1 (142172611)	1.34 (< 0.001)
	50-70	465.8 (19565630)	147.2 (6182930)	1534.9 (64466247)	1387.7 (58283317)	1611.2 (67668702)	3649 (153256355)	2.081 (< 0.001)
Overall		224.7 (9439140)	80 (3359367)	597.9 (25111930)	517.9 (21752563)	807.6 (33917787)	2901.2 (121852050)	

## Discussion

 This research provides valuable insight into HCU from complementary insurance in a large group of SM exposed survivors. Understanding HC accessibility and utilization in this population provides a chance for policymakers to identify the patterns of HCU in SM exposed survivors, as well as a basis for funding and allocating health services.

 This study shows that with increasing age, the average number of HCU per person increased significantly but there was a dramatic decrease in the number of survivors who utilized HC. In addition, with increasing age, the average annual cost of HCU increased significantly. Also, an increase in the severity of injury among SM exposed survivors paralleled a rise in HCU, and with increasing disability weight, the average annual cost of HCU per person increased considerably.

 This study indicates that the mean health/medical costs per person were US$ 807.6. The National Health Accounts of Iran in 2015 showed the per capita health costs of Iranian population at US$450.^20^ Given that the SM exposed survivors are an injured population, they require more health services and the health costs in this population were higher than the normal population.

 The findings demonstrated that most HCU was related to physician visits and medicines. In a study intended to investigate the utilization of HC by war survivors and its determinants, Mousavi et alshowed that most HC services used were related to physician visits and medications.^21^ Locker et almentioned third party financing reduces financial barriers of access to healthcare services.^22^ It has been noted that third party financing affects the number of households’ out-of-pocket payments and generally improved limited access to services; as a result, there could be an increase in the number of physicians’ visits. Hobdell et al concluded that lack of insurance coverage was associated with a reduction in visits.^23^

Table 5 compares HCU in SM exposed survivors with war survivors ^21^ and the Iranian general population.^24,25^ Compared to war survivors, SM exposed survivors used a lower amount of outpatient and paraclinical services and more inpatient services. In general, SM exposed survivor utilized 15.6 services per person per year, which is more than the general Iranian population and less than other war survivors. Regarding hospitalization costs, we found that the distribution of hospital costs was skewed and the standard deviation for the mean cost of hospitalization was relatively high. This trend normally happens in inpatient costs for several reasons. Usually, the majority of patients stay in hospital for a few days; but a small proportion of the patients might develop serious conditions that lead to a long hospital stay, transfer to critical care units and/or subsequent high costs. This trend usually leads to skewness in distribution of hospital costs as found in our study. Therefore, the reported results should be interpreted with caution. To address this issue, we also reported the median and interquartile range (IQR) of the inpatient costs.

**Table 5:** Comparing Health Care Utilization in SM Exposed Survivors with War Victims and general Iranian Population

**Annualized Health Care Utilization**			
	**War Victims** ^21^	**SM Survivors**	**General Population Annualized Health Care Utilization 2015 ** ^24,25^
Physician visit	8.9	6.14	4.9
Medication	6.6	6.52	3.6
Lab test	1.9	0.025	0.7
Imaging	0.9	0.67	0.4
Paraclinic	2.9	1.06	1.0
Outpatient	22.2	14.6	9.6
Inpatient/hospitalization	0.79	0.93	0.1
**Total**	22.8	15.6	9.7

 However, it is necessary to mention about hospitalization costs that it is possible that a small proportion of these patients are not registered by this insurance organization; and a small proportion of the registered patients might be admitted to hospitals that are not covered by this insurance organization. Therefore, it is possible that a fraction of inpatient costs has not been registered by the insurance organization and these inpatient costs have been underestimated in our study.

 The total annual number of HCU was higher in Iranian SM exposed survivors compared to the general Iranian individuals,^25,26^ but was lower compared with war survivors who were studied by Mousavi et al.^21^ This may have been caused by the different context of the studies and the fact that in this study, only complementary insurance data were used and basic insurance and out-of-pocket payments were ignored. The annual rate of outpatient and inpatient HCU in our study was higher than the general Iranian population but lower compared with Mousavi et al.^21^ Patankar and Trivedi showed that with hospitalization in public facilities, out-of-pocket payments for COPD were up to 62.3% of annual personal income compared to 50.7% for hospitalization in private facilities.^27^

 The annual rate of lab tests was lower in SM exposed war survivors compared with the Iranian general population and the study of Mousavi et al.^21,24,25,26^ The reason may be the fact that many laboratory services are covered by basic insurance and only some special and expensive lab tests are covered by complementary insurance. Although the number of laboratory services received in a year was low, the cost was pretty high. Widström and Eaton concluded that having health insurance coverage encourages people to use more and sophisticated health services, thus increasing the risk of being exposed to catastrophic health expenditure.^28^ Considering that in this study, only complementary insurance database was studied, it is necessary to survey social insurance and out-of-pocket expenditures to gain a relatively appropriate insight about the HCU of the study population.

 Among all services (without any category), the highest expenditure was related to rehabilitation (US$151.7), medical equipment (US$84.5) and medicine (US$83.3). This may be due to the fact that exposure to SM leads to chronic complications and delayed health problems^29^; so SM exposed survivors suffer from chronic health problems during the years after exposure and need rehabilitation services, considering the fact that most of these services are expensive. Therefore, not only can it impose catastrophic health expenditures on survivors and their family, but also impose great financial burden on the health system.

 The lowest expenditure pertained to nursing (US$5.2) and emergency services (US$7.5). Due to the fact that MVAF pays a monthly pension for survivors under the title of home nursing expenses according to their disability weight, they seek for nursing care in few and urgent cases. It is caused by the fact that exposure to SM leads chronic disorders, so survivors may need less emergency care.

 This study shows that with increasing age, the average number of HCU per person increases significantly. This is because of the fact that with increasing age, due to the accompanying of other chronic diseases as well as aging problems by chronic complications caused by exposure to SM, survivors need more HC and therefore utilize more services. Chang et al, aiming to determine the pattern of HCU in Taiwan’s national health insurance system, reported that there is a direct correlation between age and HCU, such that individuals who were 65 and over had the highest HCU at almost 10 times more than the lowest age group.^30^ Abera Abaerei et al showed that as age increases, the odds of HCU also increase (OR = 1.02), i.e. for a one-year increase in age, the odds of seeking HC increase by 2%.^31^

 However, with increasing age (with other variables kept constant), the chances of not using HC among survivors increases significantly. In other words, with increasing age, the number of those who utilized HC decreased significantly. The chances of not using HC for the age group of 70 + was 1.208 times higher than the base age category (< 50 years). On the other hand, with increasing age, the average annual cost of HC utilization per person increases significantly. For people in the age group of 70 + , the average annual cost of HC utilization per person was significantly 2047 times higher than the base age group (< 50 years). In other words, the average annual cost of HC utilization per person in the age group of 70 + , was 204.7% higher than the base group. This finding could indicate that although the number of people who utilize HC decreases with age, older people receive more expensive HC. Considering that in this study, only complementary insurance services were examined, it can be concluded that the elderly mostly suffer from chronic diseases and may have used the HC that were covered by basic insurance. Therefore, in the future studies, researchers should pay attention to the patterns of HCU in basic insurance as well as out-of-pocket payments. Considering that increasing age is equal to a rise in the average annual cost of HCU per person and the high expenditure of rehabilitation services, it is of crucial importance to consider the effects of aging and HC needs in SM exposed survivors. Mousavi et al concluded that enhancing the load of rehabilitation and health planning to reduce disability in war survivors would be useful to decrease the financial burden and cost of HC.^21^ Therefore, in HC planning for war survivors, special attention should be paid to the effects of aging on HCU and costs, and HC needs among elderly veterans should be considered.

 With increasing disability weight and also with increasing severity of injury in people with lung and eye injuries, the average number of HCU increases significantly, while for skin injuries, with increasing severity of skin injuries, the average number of HCU per person decreases considerably. Considering that SM has several consequences in various organs of the human body including chronic bronchitis and pulmonary fibrosis in the respiratory system^32^; dry skin, multiple cherry angiomas and hyperpigmentation in the skin^33^ and bulbar conjunctiva and limbal tissue abnormalities in the eyes,^29^ the pulmonary system and lungs are the major organs which are affected by the SM agent. Many of these complications, especially lung involvement, extend from acute to chronic (long-term and delayed phase).^34^ Therefore, considering that survivors with lung disorders are more likely to suffer from chronic complications, they need more HC. On the other hand, it has been found that for survivors with severe lung injury, the average number of HCU per person per year was 1.134 times greater than those with the “No lesions” level of severity. In other words, under the same conditions, they receive 13.4% more HC per year than the base group (No lesions). These findings indicate that survivors with severe injuries need more HC. Given that according to the results of the study, about 28% of the study population had not received any HC during one year, in the future studies, the issues of access and equality in HCU should be considered.

 With increasing severity of injury in the lungs, eyes and skin, the chance of not using services decreases. In other words, as the severity of the injury increases, people utilize more HC. For lung injuries, with increasing severity of lung injury, utilization of HC increases. Also, with increasing severity of injury in lung injuries and increasing disability weight, the average annual cost of HCU per person increases significantly. For lung disorders, the average annual cost of HCU rises significantly and survivors with severe lung disorders had significantly higher costs than others. The high rate of utilization of HC can be due to the need for more HC. On the other hand, people with severe injuries need long-term HC and also need a wide range of costly health services for treatment, nursing and rehabilitation. Thus, both victims of the chemical war and their families are harmed because of the long-term caregiving that is too expensive.

 The Iranian total health expenditure and the governmental health expenditure (GHE) were about 1 629 000 000 IR million Rials and 415 000 000 IR million Rials in 2018, respectively; therefore, the healthcare costs of the chemical survivors exposed to mustard gas (1 234 030 854 000 IR Rials), from complementary health insurance were estimated at about 0.1% of total health expenditure and 0.3%of governmental health expenditure.

 In conclusion, years after exposure to chemical warfare, the age of war survivors has increased and therefore this population is facing health problems caused by advanced aging. As a result, the health system may face an increase in the health expenditure because of aging in this large population. Therefore, health policy makers and managers should pay special attention to the effects of aging, health needs and HC costs of elderly SM exposed survivors who are suffering from chronic health problem during the years after exposure and need rehabilitation services, considering the fact that most of these services are expensive. Therefore, it can impose catastrophic health expenditure on survivors and their families, as well as a great financial burden on the health system.

 According to the results of the study, about 28% of SM exposed survivors had not received any HC during one year, in the future studies, the issues of access and equality in HCU should be considered.

###  Limitations and Strangeness

 The current study suffers from some limitations. Health care utilization and expenditure were estimated using complementary insurance only and basic insurance data and out-of-pocket payments were not studied. It is plausible that at least some survivors received health care services outside complementary insurance.

 Only a few researches have been carried out on HCU behavior and access to health-care in war survivors. The main strength of the present research is that it evaluates HCU and expenditures in a very large population-based study. Another limitation of this study is the exchange rate fluctuations and the need to pay attention to it in costs.

 Another limitation of this study is the inflation with exchange rate fluctuations and the need to pay attention to it in costs.
